# Underlying goals of advance care planning (ACP): a qualitative analysis of the literature

**DOI:** 10.1186/s12904-020-0535-1

**Published:** 2020-03-06

**Authors:** Nienke Fleuren, Marja F. I. A. Depla, Daisy J. A. Janssen, Martijn Huisman, Cees M. P. M. Hertogh

**Affiliations:** 1 General Practice & Elderly Care Medicine, Amsterdam Public Health, Amsterdam UMC, Vrije Universiteit Amsterdam, Amsterdam, The Netherlands; 2 Care and Public Health Research Institute, Department of Health Services Research, Maastricht University, Maastricht, The Netherlands; 3 CIRO, Research and Development, Horn, The Netherlands; 4 Epidemiology and Biostatistics, Amsterdam Public Health, Amsterdam UMC, Vrije Universiteit Amsterdam, Amsterdam, The Netherlands; 5 Vrije Universiteit Amsterdam, Faculty of Sociology, Amsterdam, The Netherlands

**Keywords:** Advance care planning, Goals of care, End-of-life, Quality of care, Autonomy, Relationships

## Abstract

**Background:**

Since the introduction of the concept of advance care planning (ACP), many studies have been conducted exploring beneficial effects. These studies show a heterogeneity in clinical endpoints, which reflects diversity of goals connected to ACP. This study aims to get insight in the range of underlying goals that comprise the legitimacy of ACP.

**Methods:**

Systematic literature search in PubMed, EMBASE, PsychInfo, CINAHL and Cochrane Library. Articles on normative aspects of ACP were included, based on title and abstract. Due to the quantity of inclusions, of which many had similar content, purposive sampling was used to select articles for full text document analysis. Analysis stopped once saturation was reached.

**Results:**

In total, 6497 unique articles were found of which 183 were included. Saturation was reached after document analysis of 55 articles (30%); this yielded 141 codes concerning goals of ACP and also 70 codes about objections against ACP, which shed light on the underlying goals of ACP as well. We identified five underlying goals: respecting individual patient autonomy, improving quality of care, strengthening relationships, preparing for end-of-life, reducing overtreatment.

**Conclusions:**

Five distinctive underlying goals of ACP were identified, each with corresponding objections that need to be considered. Specifying underlying goals of ACP may direct the debate on definitions, methods and preferred outcomes of ACP.

This study was funded by the Netherlands Organisation for Health Research and Development, grant 839120002.

## Background

Advance care planning (ACP) is ‘a process of communication that aims to ensure that clinical care is consistent with patients’ preferences for care’, Joan Teno and colleagues wrote in one of the first accounts of ACP in 1994. Whilst the concept of ACP was originally introduced as a means of preserving individual autonomy of patients once they become incapacitated, Teno pleaded for empirical research focused on adjusting the normative principle, ‘so that it better reflects the possibilities of real behavior’. [1]

In line with this, a recent international Delphi panel stated that: ‘The goal of advance care planning is to help ensure that people receive medical care that is consistent with their values, goals and preferences during serious and chronic illness.’ [2] Although this consensus statement is rather close to the aim that Teno described back in 1994, the concept of ACP has evolved substantially. The Delphi process showed that – despite Teno’s early account – the focus of ACP has long been on ‘eliciting treatment instructions to be used when an individual’s decisional capacity has been lost’, before it shifted towards communication about goals and preferences. Furthermore, the concept of ACP has broadened to include more patient groups and even healthy individuals [3]. In the past few decades, a variety of goals and supposed beneficial effects of ACP has been described and researched, highlighting this shift of the concept. For example, the Study to Understand Prognoses and Preferences for Outcomes and Risks of Treatments (SUPPORT, 1995) aimed ‘to improve end-of-life decision making and reduce the frequency of a mechanically supported, painful, and prolonged process of dying’ [4], whereas the European ACTION project (2016), focusing on patients with advanced lung or colorectal cancer, hypothesizes that ACP ‘improves the quality of life and reduces the symptom burden of patients’. [5] Until 2018, the Wisconsin-based ACP program Respecting Choices® stated on its website that Respecting Choices (RC) ‘helps to achieve the Triple Aim for patients who use the most health services and need the most support: RC improves patient care experience; RC improves population health; RC reduces the per capita cost of care.’ [6] Two years later, the same website asserts that lowering health care costs is not an aim of the Respecting Choices®. Instead, its aim ‘is to support person-centered decision making’. [7]

This heterogeneity in the concept and envisaged outcomes of ACP reflects diversity and development in underlying goals. Although they are not always explicitly mentioned, identifying which goals prevail is crucial in understanding both motivation and hesitation among health care professionals and patients while engaging in ACP. If not all participants are aware of the goals, do not share the same goals, or if they do not endorse these goals, they might hesitate to invest their time in ACP. Discussing and defining goals prior to development of an ACP intervention might help in reaching these goals and overcoming barriers to successful implementation.

This study aims to identify underlying goals of ACP, according to scientific literature. It focuses on empirical studies as well as contemplative articles to get insight in the range of goals that comprise the legitimacy of ACP.

## Methods

A systematic literature search was conducted in PubMed, EMBASE, PsychInfo, CINAHL, and Cochrane Library, using various search terms for ACP and goals. As we expected moral goals and values to be relatively often discussed in articles describing cultural or religious minorities, we included ‘spiritual’, ‘cross-cultural comparison’, and ‘religion’ in our broad spectrum of search terms. The search was performed on November 29, 2017. For the complete search, see Additional file 1.

The systematic literature search was performed in order to identify potentially relevant papers on goals of ACP that could be included in the critical interpretive synthesis [8]. Search results were screened for inclusion by two reviewers (variable couples) independently on title and abstract (Table 1). We included articles on normative aspects of ACP, including goals and objections, irrespective of article type including qualitative and quantitative original research, reviews, articles on legal and ethical aspects, contemplative articles, editorials etc. Inconsistencies were discussed between the two reviewers until agreement was reached.

**Table 1:** in- and exclusion criteria

*Inclusion criteria*	
About ACP as a process	
Normative aspects of ACP as a major topic	
Abstract includes at least one goal of ACP or reflects upon goals or barriers of ACP	
Publication ≥1990	
Language: English, German or Dutch	
Full text available via the library of our institution (VU and VUmc)	
*Exclusion criteria*	
About children or adolescents	
About psychiatric disorders	

Subsequently, a critical interpretive synthesis of the included papers was conducted. According to Dixon-Woods, ‘the product of the synthesis is not aggregations of data, but theory grounded in the studies included in the review.’ [8] This means that we did not simply count the goals of ACP in the included studies; as the objective of our study was to obtain a more comprehensive understanding of the range of goals that comprise the legitimacy of ACP, we used the included studies as material for a qualitative document analysis. Anticipating that after analyzing a certain amount of full text articles no more underlying goals of ACP would be identified (i.e. data saturation would be reached), we used the qualitative method of purposive sampling for full text analysis [8]. Purposive sampling is used in interview studies and describes the practice of explicitly selecting cases that are likely to generate appropriate and useful data [9]. Applying this concept, we classified the included articles according to article type (empirical; review; contemplative) and the main focus of the article (ACP in general / focus not specified; ACP for specific cultural or religious groups; ACP in specific settings or for patients with specific diseases). Combining these three options for article type and three options for main focus resulted in nine categories. Every included article was assigned to one out of these nine categories, based on title and abstract (Table 2). This was done by one researcher (NF).

**Table 2:** criteria for categorizing articles

*Type of article*	*Main focus*
1. Empirical studies including focus groups and interviews	A. Not specified
2. Reviews, both narrative and systematic	B. Culture including ethnic and religious minorities
3. Contemplative articles, for instance ethical literature, commentaries, letters to the editor	C. Disease or setting such as nursing home or operating room

For the qualitative analysis – the critical interpretive synthesis of the literature – we selected articles from the sampling frame i.e. the 183 included articles. We aimed at selecting a variety of articles, starting with 20% of each category. Within each category, we selected the top 20% of articles with the most extensive reflections upon goals or objections against ACP. This was done by two researchers (NF and MD), who independently scored all abstracts per category from 1 (low expectations about extensiveness of reflections upon goals or objections against ACP) to 5 (high expectations), based on title and abstract. We included articles which reflected on objections against ACP, as those reflections might reveal and challenge underlying goals. Subsequently, the two researchers (NF and MD) discussed their ratings and expectations per article, after which they decided on a final score. Based on the sum scores, all articles were sorted per category from most to least extensive reflections on ACP. The top 20% of each category was selected for full text document analysis. If the cut-off of 20% was between articles with the same scores, the two researchers jointly selected which article(s) were to be included.

After analysis of 20% of articles, we checked if saturation had been reached. We plotted how many codes had been added by each additional article. If the slope of the graph would be around zero, saturation was believed to be reached. If not, the next 10% of the ranked articles per category were selected by the two researchers (NF and MD) in the same manner, until saturation was reached.

Document analysis was performed on selected articles. Citations about goals of ACP and objections against ACP were coded in a constant comparative method, creating provisional categories and defining their boundaries increasingly precise along the way, resulting in meaningful categories. This was done in ATLAS.ti version 7.5.16 (ATLAS.ti GmbH, Berlin) by one researcher (NF).

Codes were organized in a code tree, which was made and adapted from the third article onwards, and was discussed between two researchers (NF and MD). After analyzing 10% of the articles, all codes were discussed within the research team. After analyzing 20%, we inductively identified sets of goals. Within each set, all goals were consistent with each other and did not conflict. Each set was covered by a distinctive underlying goal of ACP. The classification and designation of underlying goals were extensively discussed within the research team.

## Results

**Fig. 1:**
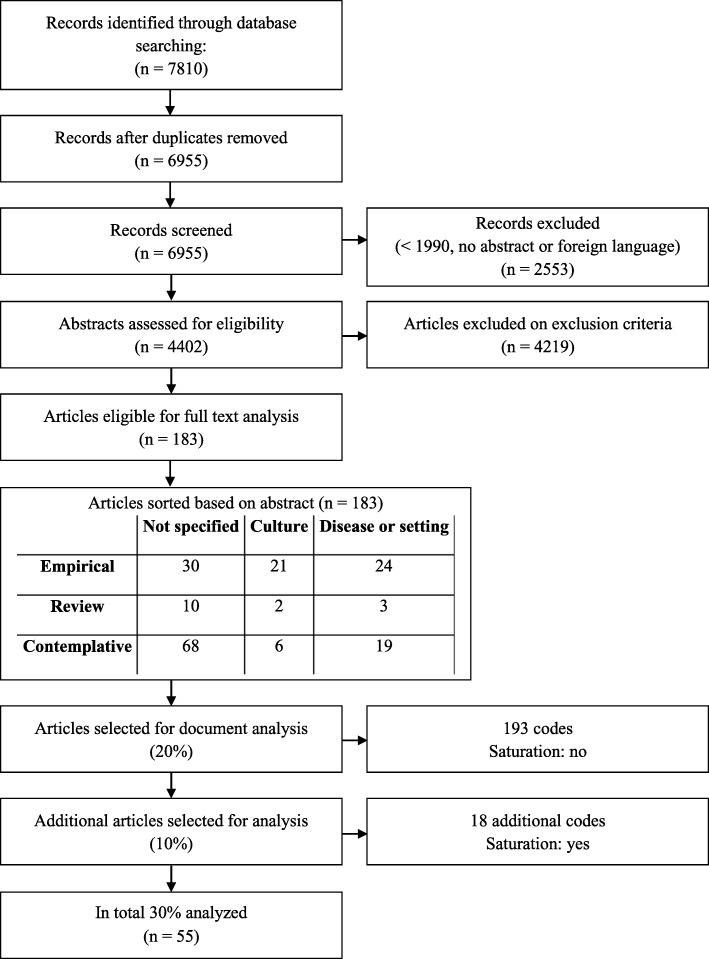
Flow chart for literature search and selection of articles

**Fig. 2:**
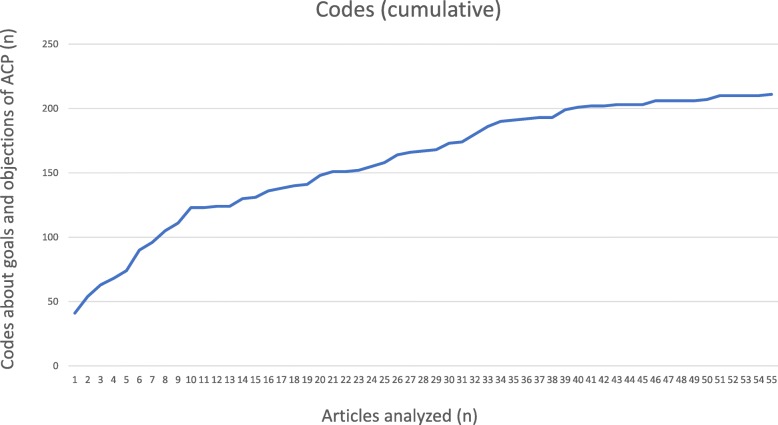
Cumulative amount of codes about goals and objections of ACP during the analyzing process

Among 4402 abstracts screened, 183 articles were eligible for full text document analysis. In the first round of qualitative document analysis (37 articles; 20%), 130 codes for goals of ACP and 63 codes for objections against ACP were found. As saturation had not been reached at this stage, another 18 articles were analyzed, resulting in 18 additional codes, of which 11 about goals and 7 about objections of ACP (Fig. 1 and Additional file 2). Fig. 2 shows that saturation had been reached at this point. Characteristics of the included articles are listed in Table 3.

**Table 3:** Included articles, analyzed in rounds 1 and 2

First round (37 articles, 20%)	Category	Article type	Study characteristics
1 Kolarik 2002 [10]	Contemplative, focus not specified	Conceptual paper	List of objectives for ACP: patient objectives, family or surrogate objectives, health care provider objectives, desirable characteristics of the process.
2 Hackler 2004 [11]	Idem	Ethical paper	Exploration of two kinds of justifications for advance directives, with two case descriptions.
3 Rietjens 2016 [12]	Idem	Editorial	Editorial for special issue about ACP.
4 Aitken 1999 [13]	Idem	Overview	Overview article about ACP for family physicians.
5 Hammes 2001 [14]	Idem	Editorial	Editorial.
6 Perkins 2007 [15]	Idem	Perspective	Perspective on the role of advance directives.
7 Schicktanz 2009 [16]	Idem	Ethical paper	Ethical considerations of the interplay between personal and cultural identity in interpreting advance directives.
8 Sudore 2010 [17]	Idem	Perspective	Perspective on the objective of ACP.
9 Robins-Browne 2014 [18]	Idem	Ethical paper	Ethical considerations about the complexities of ACP.
10 Johnson 2017 [19]	Idem	Ethical paper	Ethical considerations about the role of ACP in end-of-life care.
11 Van Delden 2017 [20]	Idem	Commentary	Commentary on the PREPARE-trial.
12 Vogel 2011 [21]	Idem	News item	News about the Canadian framework for ACP.
13 Wheatley 2015 [22]	Idem	Ethical paper	Ethical issues in palliative care, including ACP.
14 Song 2016 [23]	Empirical, culture-focused	RCT	ACP intervention ‘SPIRIT’ vs. usual care. Subjects: 210 dyads of patients on dialysis and their surrogates; subgroup analysis of African Americans vs. whites. Outcome measures: dyad congruence on goals of care, surrogate decision-making confidence, patient decisional conflict, surrogate anxiety, surrogate depression, surrogate post-traumatic distress symptoms.
15 Asai 1997 [24]	Idem	Focus group	Focus group on life-sustaining treatments for terminally ill patients and attitudes towards advance directives and possible barriers to using them in the clinical setting. Subjects: 7 specialists in internal medicine from Japan.
16 Perkins 2002 [25]	Idem	Interview study	Interviews about cultural attitudes influcancing decisions whether to perform ACP. Subjects: a purposive sample of 26 Mexican-American, 18 Euro-American, and 14 African-American inpatients in Texas, USA.
17 Ko 2012 [26]	Idem	Focus groups	Focus group and interview study to explore knowledge, attitudes, and behavior about advance directives in Korean Americans. Subjects: 23 Korean Americans.
18 Wicher 2012 [27]	Review, culture-focused	Systematic review	Systematic review of 46 studies examining African American preferences related to end-of-life care and decision making.
19 Singer 1998 [28]	Empirical, focus not specified	Interview study	Interviews about the purpose of ACP. Subjects: 48 patients on hemodialysis.
20 Robinson 2011 [29]	Idem	Interview study	Interviews about the applicability and usefulness of an ACP intervention and the ACP process. Subjects: 9 dyads of patients with advanced lung cancer and a family member, who participated in an ACP intervention.
21 Jeong 2012 [30]	Idem	Interview study	Interviews based on the ‘Values Clarification Worksheet’. Subjects: 3 residents living in an residential aged care facility in Australia, 11 family members, and 13 registered nurses.
22 Sudore 2017 [31]	Idem	Delphi study	Delphi study on the definition of ACP.
23 Rhee 2013 [32]	Idem	Interview study	Interviews on the impact of ACP on interpersonal relationships. Subjects: 17 general practitioners in Australia.
24 Michael 2017 [33]	Idem	Focus groups	Focus groups exploring awareness, attitudes, and experiences of ACP. Subjects: 15 community dwelling older people and 27 unrelated offspring/caregivers of older people.
25 Martin 1999 [34]	Empirical, disease-focused	Interview study	Interviews about experiences with and opinions about ACP. Subjects: 140 patients with HIV/AIDS who had participated in an ACP trial.
26 Johnson 2017 [35]	Idem	Interview study	Interviews about patient autonomy and ACP. Subjects: 11 consultant oncologists and 7 palliative medicine doctors.
27 Loggers 2014 [36]	Idem	Mixed methods study with survey (quantitative) and interview (qualitative) part	Survey and interview about experiences with ACP. Subjects: 18 patients after hematopoietic cell transplants and 11 bereaved caregivers of patients who had died after hematopoietic cell transplant. Outcome measures (quantitative): having a living will; having a formally designated proxy; having discussed some aspect of ACP with family/friend prior to transplant; perceptions of the value of ACP; having discussed mortality risk with the medical team pre transplantation; hope; medical team’s commitment.
28 Thoresen 2016 [37]	Idem	Participant observation and interviews	Participant observation of ACP conversations, followed by interviews. Subjects: 7 nursing home patients in 7 different nursing homes in Norway and the relatives who joined the ACP conversation.
29 Sellars 2017 [38]	Idem	Interview study	Interviews about patient and caregiver perspectives on ACP. Subjects: 24 patients with end stage renal disease and their caregivers (*n* = 15).
30 Johnson 2016 [39]	Review, disease-focused	Systematic review with thematic analysis	Systematic review of perceptions and experiences on ACP of cancer patients, their families, and health care providers. Thematic analysis was performed on the included studies: 19 quantitative studies, 17 qualitative studies, 4 mixed methods studies.
31 Mehlis 2016 [40]	Review, focus not specified	Ethical and legal perspective	Ethical and legal perspective on self-determination and the risk of overtreatment, with a focus on ACP.
32 Llewellyn 2017 [41]	Idem	Interview study	Interviews about death and dying and reflections on the effect of these conversations. Subjects: 21 healthy adults 54–65 years old.
33 Sanchez-Gonzalez 1997 [42]	Contemplative, culture-focused	Medical history perspective	Historical perspective on the emergence of advance directives in the USA, as compared to Europe. Focus on cultural differences.
34 Schmidt 2017 [43]	Contemplative, disease-focused	Overview	Overview article about incorporating ACP on hemodialysis units.
35 Holley 2005 [44]	Idem	Perspective	Perspective on the timing, purpose, and effect of ACP in patients with end-stage renal disease.
36 Gastmans 2010 [45]	Idem	Ethical perspective	Ethical perspective on euthanasia and advance euthanasia directives for patients with severe dementia.
37 Holley 2012 [46]	Idem	Overview	Overview article on the role and timing of ACP for patients with chronic kidney disease and end-stage renal disease.
**Second round (18 articles, 10%)**	**Category**	**Article type**	**Study characteristics**
38 Schwartz 2002 [47]	Empirical, focus not specified	Randomized controlled trial (pilot study)	Pilot randomized controlled trial of an ACP intervention. Subjects: 61 ambulatory geriatric patients. Intervention: ACP discussion with a trained nurse facilitator and documentation of patient goals and preferences. Control: Massachusetts Health Care Proxy form. Outcome measures: knowledge of ACP; treatment preferences (and congruence between patient and health care agents; response shifts in values; quality of life.
39 Seymour 2004 [48]	Idem	Focus groups	Focus groups on advance care statements. Subjects: 32 older people or their representatives.
40 Cornally 2015 [49]	Idem	Focus groups	Focus groups on the implementation of the ‘Let me decide’ ACP program. Subjects: 15 clinical nurse managers and 2 directors of nursing in long term care facilities where the ‘Let me decide’ ACP program had been implemented.
41 Stanford 2013 [50]	Empirical, culture-focused	Focus groups	Focus group study on the perceived relevance of ACP in Knysna, South Africa. Subjects: 51 participants including pastors, hospice staff, teachers, and community caregivers in Knysna, South Africa.
42 Lee 2016 [51]	Idem	Interview study	Interview study on signing one’s own do-not-resuscitate directive among older nursing home residents in Taiwan. Subjects: 11 older nursing home residents from Taiwan.
43 Zientek 2006 [52]	Contemplative, culture-focused	Ethical perspective	Ethical considerations regarding end-of-life care and the role of advance directives in Texas, and their impact on Roman Catholic health care providers.
44 Prendergast 2001 [53]	Contemplative, disease-focused	Overview, historical perspective	Historical overview of the development of ACP in the first decade since the Patient Self-Determination Act, with a focus on its importance for intensive care units.
45 Kuhlmann 2016 [54]	Idem	Overview, historical perspective	Overview of the development and importance of ACP for patients with end-stage renal disease.
46 Drought 2002 [55]	Review, focus not specified	Review and ethnographic study	Literature review on the role of choice in end-of-life decision making, followed by an ethnographic study. Subjects: 88 terminally ill patients with solid tumor cancer or AIDS who were followed longitudinally, together with 1–3 of their family members or friends, and 2 of their outpatient clinic providers.
47 Robertson 1991 [56]	Contemplative, focus not specified	Opinion	Opinion article on the role of advance directives.
48 Levinsky 1996 [57]	Idem	Opinion	Opinion article on the purpose of advance medical planning.
49 Darr 1996 [58]	Idem	Overview	Overview article on the use of advance directives.
50 Davis 2002 [59]	Idem	Ethical perspective	Ethical perspective on the concept of precedent autonomy in advance directives.
51 Edwards 2011 [60]	Idem	Ethical perspective	Ethical perspective on the concept of precedent autonomy and the theory of ‘the other self’ in relation to advance directives.
52 Ahluwalia 2012 [61]	Idem	Letter to the editor	Comment stating that an earlier article wrongfully used a too narrow definition of ACP.
53 Wolff 2012 [62]	Idem	Ethical perspective	Ethical perspective on the role of autonomy, quality of life, and advance directives in end-of-life decision making for patients with dementia.
54 Davison 2006 [63]	Empirical, disease-focused	Interview study	Interviews on the role of hope in the context of ACP. Subjects: 19 patients with end-stage renal disease.
55 Piers 2013 [64]	Idem	Interview study	Interviews on the views of older people on ACP. Subjects: 38 older people (71–104 years old) with limited prognosis due to malignancy, organ failure, or frailty.

### Underlying goals of ACP

The 141 codes about goals and 70 codes about objections were classified into five underlying goals of ACP. These underlying goals are: respecting individual patient autonomy, improving quality of care, strengthening relationships, preparing for end-of-life, and reducing overtreatment.

### Respecting individual patient autonomy

With this underlying goal, individual patient autonomy is considered to be at the basis of ACP. The process of ACP is meant to extend individual autonomy to stages of decisional incapacity.

‘*Advance care planning is generally championed as a means by which competent patients can extend their involvement in and control of decisions regarding their own health care beyond the point at which they lose capacity as a result of illness or injury.*’ [art.30] [39].

According to this underlying goal, ACP supports that health care decisions will be based upon patient preferences. Treatments will be aligned with those preferences. The process of ACP increases patients’ sense of control. Besides, it shifts responsibility from family members and health care professionals to patients. In this way, ACP also protects health care professionals against liability risks and claims of relatives with different opinions on what the patient would have wanted: they might use ACP documentation as evidence that treatment decisions were made according to patient preferences instead of the health care providers’ own ideas.

‘*ACP may also provide the health care provider proof against angry, distant relatives about what the patient really wanted.*’ [art. 1] [10].

Focussing on individual patient autonomy, ACP is acknowledged as an advancement of the practice of advance directives (ADs), which was introduced in the 1990s [53]. Although respect for patient autonomy – and even extending it to stages of incapacity – has been generally accepted, implementation of ADs proved to be difficult. Critics emphasized that preferences might change over time or with progression of disease. ACP is thought to overcome most of these difficulties, as it is a continuous process of defining, reconsidering, and documenting preferences. Also, ACP might improve the applicability of ADs, by specifying how the AD is to be used.

‘*ACP should clarify whether decisions are to be made by consensus of the family or by one person only, and the amount of leeway they have when interpreting preferences.*’[art. 1] [10].

In the end, ACP helps the patient to get as close as one might get to full self-determination. While respecting individual patient autonomy, one places the personal goals and values of the competent patient central. The actions of both family members and caregivers are directed by the patient through written or oral advance statements. The question remains if even the future incompetent self – for example the demented patient – should be subordinated to the values she formerly held, a concept known as ‘precedent autonomy’.

*Similarly, one may argue that an advance directive should be honored because what the patient earlier said she wanted is what she truly wants now even though she is too incapacitated to know what she wants now.* [art. 50] [59].

If the underlying goal of respecting individual patient autonomy prevails, precedent autonomy tends to be higher valued than the current interests of the patient. This distinguishes this underlying goal from other goals, in which formerly held values play a less important role. The underlying goal of respecting individual patient autonomy is the only one in which pre-defined preferences (or, if not available, reconstructions of what the patient would have wanted) outweigh the current best interest of the patient (what would be ‘best’ for the patient in the current situation).

### Improving quality of care

According to the underlying goal of improving quality of care, ACP is a means of tailoring care to the patients’ needs, especially at the end of life. Unlike the underlying goal of respecting individual patient autonomy, with this goal, patient needs are more important than patient preferences. Tailoring treatment decisions to patient needs means striving to integrate professional standards and patient preferences.

*Preparing patients for such decisions shifts the focus away from premature treatment decisions based on incomplete or hypothetical information and ensures that complex health care decisions are based on a more comprehensive set of considerations, including the current clinical context, shifting and evolving goals, and patients’ and surrogates’ needs.* [art. 8] [17].

Thus, shared decision making is a prerequisite for patient-centred care, and ACP is a means of improving patient participation in shared decision making. For patients without decision making capacity, this means that health care professionals try to find out what values the patient holds and what preferences they used to have, so these preferences can be integrated in clinical decision making. Ideally, this process starts before the patient loses decision making capacity.

For patients with decision making capacity, this means being prepared for in-the-moment decision making. Through ACP, patients will be informed about their illness and know about their prognosis, enabling health care professionals to prepare patients for conscious end-of-life decision making.

‘*The outcomes will, of course, often still be sad and painful, but the process will ensure that the key values relevant to the context have been adequately considered.*’ [art. 9] [18].

The underlying goal of improving quality of care supposes that timely discussions about preferences and prognosis, will make patients receptive to palliative care options earlier in their illness trajectory. This enables the health care professional to initiate palliative care in time.

‘*ACP assists clinicians challenged by treatment cessation decisions, particularly in OP [older persons] where extended life expectancies may be associated with co-morbidities and increasing frailty.*’ [art. 24] [33].

Jointly, patients or their surrogate decision makers, family members and health care professionals take health care decisions that are in the best interest of the patient. Quotes like ‘improving end-of-life care’ and ‘a good death’ were frequently found, which means that there is a supposed consensus about what ‘high-quality end-of-life care’ or ‘a good death’ is.

‘*A “good death” contributes to organizational maintenance, while ‘bad’ deaths drain resources and create emotional distress in staff and families. ACP, therefore, may benefit not only patients with terminal illness but also the healthcare institution by systematizing and encouraging “dying well”.*’ [art. 26] [35].

With the underlying goal of improving quality of care, health care professionals have an interest in ACP. Because health care professionals need to know patient preferences for care, it remains usually the health care professional who determines what subjects are to be discussed, at which moments these discussions should occur, and what options there are. This enables them to provide good quality care.

At the end of life, patients are vulnerable to being influenced by others. In reaction to potential threats to the patients’ wellbeing, health care professionals feel the urge to stand up for their patients. ACP helps them to define what would be best for a particular patient in a particular situation. This also reduces the burden on health care professionals

‘*Staff felt they were now in a position to provide care that was largely based on the wishes of residents. For many this created a sense of “knowing how to care”.*’ [art. 40] [49].

### Strengthening relationships

With this underlying goal, ACP is neither primarily about autonomy nor about decisions. Outcomes in terms of chosen treatments are less important than the harmony of the process. Through discussions about presently held values as well as preferences for future care, families and health care professionals enhance their commitment to the patient.

‘*Patients recognize that they cannot anticipate all the possible circumstances of dying. However, they can direct the people who have cared for them in their lives to continue to care for them as they die.*’ [art. 44] [53].

Especially at the end of life, patients want others to support them. This will relieve the burden of illness and physical decline. It enables family and friends to show their empathy with the patient.

‘*Patients also used the interview to acknowledge the closeness and importance of the relationship with their participating family members*.’ [art. 20] [29].

Besides, ACP empowers families in standing up for the patient, enhancing relational autonomy and experience more agreement about goals of care.

‘*The participants appreciated the positive impacts that ACP could have on interpersonal relationships, such as enhancing patient-family relationships, helping resolve conflicts between families and health professionals and improving trust and understanding between patients and health professionals.*’ [art. 23] [32].

### Preparing for end-of-life

With this underlying goal, ACP is a means of preparation for, and of coming to terms with end-of-life. Unlike the underlying goal of individual autonomy, this underlying goal focuses on finding meaning and peace of mind, which can be supported by discussing the future.

‘*ACP facilitated the old person to continue to realise ‘the essence of their being’, to experience ‘gerotranscendence’ in end-of-life moments, and to die in a way consistent with ‘the essence of their being’, as they wished.*’ [art. 21] [30].

Discussions focus on personal values and concerns rather than on care decisions. In this way, both patients and their loved ones feel able to cope and experience a relief of anxiety, depression and burden.

*‘One participant commented on the way in which ACP helped him to consider the manner of his death and the way in which that would affect his children. He concluded that confronting and planning for his death were important steps in helping him and his children cope.’* [art. 25] [34].

This provides the opportunity to accept the prospect of death and prepare emotionally for death and dying. If the patient prefers to avoid discussions about resuscitation or other life-sustaining treatment, according to the underlying goal of preparing for end-of-life, those issues should not be discussed.

‘*Most older persons in this study wanted to plan for or control the end-of-life stage, but only for those issues made important to them in their own experiences or fears.*’ [art. 55] [64].

### Reducing overtreatment

With this underlying goal, ACP is a means of defining value for money at the end of life. Its focus on medical interventions places this underlying goal close to improving quality of care. With both goals, the judgement of health care professionals is important. This judgement defines the reasonable options that the patients is allowed to consider. However, if reducing overtreatment is the main underlying goal, the outcome is defined in terms of health care use and the extent to which resources are used efficiently.

‘*It is clear that attempting CPR [cardiopulmonary resuscitation] would be physiologically futile for some patients. It might also cause psychological distress to others, lead to an undignified death and deflect the cardiac arrest team away from other patients.*’ [art. 13] [22].

Reducing overtreatment is attractive to policy makers, because from an economics perspective, it is assumed that ACP will reduce cost without reducing quality of life.

*‘A further concern may be that financial resources not be squandered in prolonging a life of very low quality. ( … ) Decisions to forgo costly treatment that has little benefit at the end of life should be easier if the patient has specifically declined it.’* [art. 2] [11].

Through ACP, limited resources will be used appropriately, while reducing costs for patients as well as for society.

*‘Even when the purpose is not cost containment, there may be a philosophical emphasis on limiting, rather than maintaining, treatment. For example, one physician described advance planning as a method to “avoid excessive and undesired interventions in the final years of life.”’* [art. 48] [57].

*‘Patients who had discussions with their physicians about end-of-life planning, received less aggressive medical care at the end of life and were transferred to hospice care earlier.’* [‘*Die Patienten, die Gespräche mit ihren Ärzten über die Planung der letzten Lebensphase geführt hatten, erhielten weniger aggressive medizinische Behandlung vor dem Tod und wurden früher ins Hospiz verlegt.*’] [art. 31] [40].

### Objections

For each underlying goal that we identified, specific objections were identified as well. These objections are to be seen as risks of each particular underlying goal of ACP.

The main objection against the autonomy goal is that it may suggest more control than is possible, because future circumstances and preferences are unpredictable and often much more complicated than anticipated.

*‘However, broad values statements, such as wanting to maintain dignity or be free from pain, are often too general to inform individual treatment decisions. Even specific treatment preferences may be difficult to extrapolate to specific clinical situations.’* [art. 8] [17].

Treatment decisions may be based on preferences that the patient formerly held, without knowing if the patient would still hold them in current circumstances. Besides, following (formerly held) preferences, might contravene the interests of the patient. Another objection against the underlying goal of individual patient autonomy is that it denies that decision making is engaged and emotional, rather than rational.

*‘Perhaps the greatest problem with the choice model is that it assumes that we can accept and choose our death long before the inevitability and reality of our dying becomes apparent. In other words, it proposes that rational and cognitive ways of knowing can subordinate our emotional, psychological, and embodied experiences.’* [art. 46] [55].

The main objection against the underlying goal of improving quality of care, is that it may become a moral imperative for both patients and health care professionals to be involved in ACP discussions. After all, if the patient does not express any preferences, it would be impossible for health care professionals to deliver high-quality patient-centered care, which has become the norm.

*‘It is claimed by some that involvement in ACP has already come to be seen as a ‘moral imperative’. Such an understanding of ACP would make it difficult for those who do not wish to participate to resist doing so.’* [art. 9] [18].

Another controversial aspect of the underlying goal of improving quality of care, is that ACP primarily seems to serve health care professionals and the health care system. ACP makes it easier for physicians to make health care decisions, both practically and emotionally, because part of the responsibility is shifted to the patient. Also, the patient is supposed to be better prepared through ACP, which makes it easier for health care professionals to deal with deterioration. As one health care professional put it:

*‘“Sometimes I think it’s just to make us ourselves feel better, that we’ve ticked all the boxes, that we’ve made it very clear to the patient where things are at, so that, you know, ( … ) if the family or the patient does, I mean, deteriorate and they seem to be taken by surprise by it we can, sort of, feel like we’ve almost as if we’ve said, well, I told you so.’”* [art. 26] [35].

The underlying goal of strengthening relationships has been criticized for serving the needs of family members more than patients’ needs and individual patient autonomy. Some older people fear that their children might take control too early or financially abuse their parents. Thus, if the underlying goal of ACP is strengthening relationships, one should be aware that the patient might be overlooked.

*‘( … ) ACP can be beneficial for residents’ relatives, as it may ease* their *decision making. Whereas relatives’ wellbeing and confidence in their role as surrogate decision maker may be important outcomes of ACP, the patient’s values and preferences should be central.’* [art. 3] [12].

The main objection against the underlying goal of preparing for end-of-life, is that it holds the risk that ACP is regarded as a panacea. Although ACP discussions might ease patients and their loved-ones, health care professionals should be aware that improving satisfaction and quality of life comprises more than providing ACP.

*‘Likewise, another review of the elements of EOLC [end-of-life care] that patients ranked as being important showed that quality EOLC is not simply a matter of preference expression or control over decision-making, but a complex amalgamation of: effective communication; shared decision-making; expert care; respect and compassion; trust and confidence in clinicians; an adequate environment for care; and strategies that minimise the burden on families.’* [art. 10] [19].

The underlying goal of reducing overtreatment contains the risk that ACP processes are regarded as ‘death panels’, limiting treatments for certain patients or patient groups.

*‘Some patients and caregivers, including those who had completed ACP, had initially interpreted ACP to signify death and defeat, and some became “disturbed” by ACP because they believed it was being used as a mechanism “to knock patients off.”’* [art. 29] [38].

Besides, some patients prefer to rely on God or to accept the mandate of nature. They do not want their treatments to be limited by medical doctors. If reducing overtreatment is the underlying goal, patient interest to get involved in ACP might diminish. It might even lead to distrust in health care professionals and the health care system.

*‘Pressure to transform clinicians from knowledgeable advisors to advocates for the limitation of care threatens to undermine the ethical validity of advance planning.’* [art. 48] [57].

Another concern is that seniors and patients in unfavorable conditions will experience societal pressure to forego intensive and expensive treatments.

*‘If a right to die becomes a duty to die, the living will and its progeny, the natural death act declaration, will have become a Frankenstein monster.’* [art. 49] [58].

An overview of underlying goals and corresponding objections is given in Table 4.

**Table 4:** Underlying goals of ACP and corresponding objections

Underlying goal	Objections
Respecting individual patient autonomy	Promises more control than is possible; ignores current interests of the patient; denies that decision making is engaged and emotional
Improving quality of care	Risk of ACP to be regarded as moral imperative
Strengthening relationships	Shifts focus away from the patient; risk that children take over too early
Preparing for end-of-life	Risk of ACP to be regarded as panacea
Reducing overtreatment	Pressure to refuse treatment; distrust of the health care system; against nature

## Discussion

This qualitative analysis of literature has explored underlying goals of advance care planning (ACP). It shows that, within scientific literature, five different underlying goals of ACP prevail. These underlying goals each comprise a set of goals that are consistent with each other and can be strived for at the same time. The underlying goals are: respecting individual patient autonomy, improving quality of care, strengthening relationships, preparing for end-of-life, and reducing overtreatment.

In clinical practice, one might strive for each of these underlying goals of ACP at the same time. It might get difficult however, if the patient insists on treatments that are not considered good quality of care by the health care providers, or if engaging family members would undermine patient autonomy. In these cases, ACP requires balancing the underlying goals and deciding which underlying goal prevails. Especially in those situations, it is important that stakeholders are aware of the different underlying goals that they might strive for with ACP. An open discussion between stakeholders (patient, surrogate, other relatives, health care provider) might clarify the relative value that they assign to each of the underlying goals, making it easier to reach consensus in the ACP process itself.

ACP has evolved in response to the difficulties of a rigid implementation of advance directives (ADs). However, the advancement from ADs to an ACP process did not take away all of the difficulties. ‘( …) if it turns out that few patients engage in advance care planning, this may indicate that the full range of moral concerns of patients is not captured by the present practice of advance directives or by the underlying principle of patient self-determination,’ Joan Teno wrote back in 1994. ‘Here, ethical analysis and empirical research must inform each other if our understanding is to be advanced.’ [1] With this qualitative analysis of the literature, we confirmed that a range of moral concerns is at issue, next to patient self-determination. We augmented this understanding, identifying the underlying goals that are embraced by scientists, patients, and health care professionals whose views have been published in biomedical literature.

As we studied only a selection of articles from the extensive literature on ACP, counting the frequency of codes over time would not be informative. However, freely allocating one prevailing underlying goal of ACP to each article, it seemed to us that the underlying goals of respecting individual patient autonomy and improving quality of care prevailed in earlier years, whereas the underlying goals of improving quality of care and preparing for end-of-life were more common in recent articles. Nearly all articles which focused on specific patient groups or specific settings tended to consider ACP as a means of preparing for end-of-life. On the other hand, articles about patients from specific cultural, ethnic or religious groups, commonly challenged the assumption that respecting individual patient autonomy would be the main goal of ACP, suggesting that this goal might particularly appeal to white Americans. Further research is needed to verify if these are genuine trends.

Evaluating the five different underlying goals, one might suggest that there are in fact five distinctive variants of ACP, each with its own definition, methods, and goals. Within each variant of ACP, the interests of patients, family members, health care professionals, and society will be balanced differently. If the main underlying goal is respecting individual patient autonomy, the customer is always right. However, some health care professionals would like to take clinical circumstances and their professional standards into consideration as well, aiming at improving quality of care by providing patient-centred care. At the other hand, some patients might not be interested in this health care centred goal or fear that ACP becomes a tick-box. Aiming at preparation for end-of-life, they use ACP to discuss personal fears and values, and to be seen as a person. Some patients and their loved-ones might use ACP to show their commitment to each other, aiming at strengthening relationships. Likewise, society might have an interest, using ACP to reduce overtreatment.

In cases where the interests of all stakeholders are similar, ACP might accomplish every single underlying goal. However, in case the interests of different stakeholders conflict with each other, it might not be possible to reach all underlying goals. This might explain the difficulties in reaching consensus about one single definition, and determining universal outcome measures for ACP [3, 65].

It might be helpful for patients, health care professionals, and researchers to be clear about which variant of ACP is pursued and which of the underlying goals prevails. In this way, it would be easier to reach consensus about goals of the ACP process and to identify methods for achieving those goals as well as outcome assessment.

Additionally, specifying the underlying goals of ACP in clinical settings will help to recognize and anticipate common risks associated with that particular approach of ACP. For example, if improving quality of care would be chosen as main underlying goal, the health care professional should be aware of the possibility that the patient might experience a moral pressure to keep taking part in discussions that (s)he would prefer to avoid. Weighing the potential benefits and harms, health care professionals and patients may arrive at a shared understanding of which variant of ACP would best suit their situation. This will probably make ACP more effective, or at least make patients and health care professionals less reluctant to start the conversation about this important topic.

### Strengths and limitations

Our study shows which goals of ACP have been mentioned in scientific literature. This was based on a systematic search, which yielded ethical literature, contemplative articles, and empirical studies. We used the method of purposive sampling to make an adequate selection for further analysis and we proceeded until saturation was reached.

Scientific publications are particularly useful in studying the scientific discourse, because the phrasing is generally discussed between authors and reviewed by peers before publication. Therefore, one might expect published formulations to be a genuine representation of phrases that are accepted in the scientific debate. Moreover, because of word count limits, the density of information is high. In coding citations, we kept close to the original text, which resulted in many codes with slightly different meanings. By doing so, it was possible to distinguish different underlying goals of ACP, with their corresponding objections.

The main limitation of our study was the more or less subjective inclusion and selection of articles. Because of the heterogeneity of articles, it was difficult to find strict rules for inclusion and exclusion. For example, defining if normative aspects of ACP were ‘the major topic’ of an article, based on title and abstract, was not an easy task. By doing this with two researchers and discussing every article that one of us wanted to include, we tried to reduce this subjectivity.

The same applies to the selection of articles for full text analysis. This was done by two researchers, who discussed their expectations, based on title and abstract. However, we expect that other researchers would have selected roughly the same articles, as these were considered key publications. In the end, we assume that the partly selective inclusion and selection of articles did not influence the final results of our study, as we found many different codes and continued until saturation was reached. The distinction between the five underlying goals was made after analysis of 20% (37 articles) and did not substantially change after another 10% (18 articles).

## Conclusions

Within scientific literature, five different underlying goals of ACP prevail: respecting individual patient autonomy, improving quality of care, strengthening relationships, preparing for end-of-life, and reducing overtreatment. Negotiating which goal prevails in a specific situation, might illuminate which outcomes are in accordance with the chosen goal and which risks need to be considered. Specifying underlying goals may direct the debate on definitions, methods and preferred outcomes of ACP.

## Supplementary information

**Additional file 1:.** Search terms.

**Additional file 2:.** Codes per underlying goal.

## Data Availability

The data and analysis files of the current study are available from the corresponding author on reasonable request.

## Abbreviations

ACP: Advance care planning
AD: Advance directive
