# Supplementary material for: Underlying goals of advance care planning (ACP): a qualitative analysis of the literature
Source: BMC Palliat Care. 2020 Mar 6;19:27. doi: 10.1186/s12904-020-0535-1 (PMC7059342; doi:10.1186/s12904-020-0535-1)
Supplement: Supplementary file 1 — Additional file 1:. Search terms. [file 12904_2020_535_MOESM1_ESM.docx]

**Additional file 1: search terms**

**Pubmed and Cochrane:**

("Patient Care Planning"[Mesh] OR “Advance Care Planning"[Mesh] OR "Advance Care Planning"[tiab] OR “Advance health care planning”[tiab] OR “Advance medical planning”[tiab] OR “Patient care planning”[tiab] OR “Advanced medical planning”[tiab] OR "Advanced Care Planning"[tiab] OR “Advanced health care planning”[tiab])

AND

(“Ethics”[Mesh] OR “Spirituality”[Mesh] OR “Religion”[Mesh] OR “Ethics”[tiab] OR “Ethical issues”[tiab] OR “Ethical issue”[tiab] OR “Moral policy”[tiab] OR “Moral policies”[tiab] OR “Ethical”[tiab] OR “Religiosity”[tiab] OR “Religion”[tiab] OR “Spirituality”[tiab] OR “Spiritual”[tiab] OR “Cross-Cultural Comparison”[Mesh] OR “Cross-Cultural Comparison”[tiab] OR “Cross-Cultural Comparisons”[tiab] OR “Cultural Characteristics”[Mesh] OR “Cultural Characteristics”[tiab] OR “Cultural Diversity”[Mesh] OR “Cultural Diversity”[tiab] OR “Cultural Competency”[Mesh] OR “Cultural Competency”[tiab] OR “Culture”[Mesh] OR “Culture”[tiab] OR “Goals”[MesH])

**Embase:**

(‘patient care planning’/exp OR ‘advance care planning’/exp OR ‘advance care planning’:ab,ti OR ‘advance health care planning’:ab,ti OR ‘advance medical planning’:ab,ti OR ‘patient care planning’:ab,ti OR ‘advanced medical planning’:ab,ti OR ‘advanced care planning’:ab,ti OR ‘advanced health care planning’:ab,ti)

AND

(‘ethics’/exp OR ‘religion’/exp OR ‘ethics’:ab,ti OR ‘ethical issues’:ab,ti OR ‘ethical issue’:ab,ti OR ‘moral policy’:ab,ti OR ‘moral policies’:ab,ti OR ‘ethical’:ab,ti OR ‘religiosity’:ab,ti OR ‘religion’:ab,ti OR ‘spirituality’:ab,ti OR ‘spiritual’:ab,ti OR ‘cultural factor’/exp OR ‘cross-cultural comparison’:ab,ti OR ‘cross-cultural comparisons’:ab,ti OR ‘cultural characteristics’:ab,ti OR ‘cultural diversity’/exp OR ‘cultural diversity’:ab,ti OR ‘cultural competence’/exp OR ‘cultural competency’:ab,ti OR ‘culture’:ab,ti OR ‘goal setting’/exp)

#1 AND #2 AND [embase]/lim

NOTE: MeSH term ‘Spirituality’ were replaced by ‘Religion’, which was already a search term. MeSH terms “Cross-Cultural Comparison” and “Cultural Characteristics” and “Culture” were replaced by Emtree ‘cultural factor’. MeSH-term “Cultural Competency” was replaced by Emtree ‘cultural competence’. Emtree does not have a term for ‘goals’, except for ‘motivation’, but that does not resemble moral goals, so we only used the candidate term ‘goal setting’.

**PsychInfo:**

(MA patient care planning OR MA advance care planning OR TI advance care planning OR AB advance care planning OR TI advance health care planning OR AB advance health care planning OR TI advance medical planning OR AB advance medical planning OR TI patient care planning OR AB patient care planning OR TI advanced medical planning OR AB advanced medical planning OR TI advanced care planning OR AB advanced care planning OR TI advanced health care planning OR AB advanced health care planning)

AND

(MA spirituality OR MA religion OR TI ethics OR AB ethics OR TI ethical issues OR AB ethical issues OR TI ethical issue OR AB ethical issue OR TI moral policy OR AB moral policy OR TI moral policies OR AB moral policies OR TI ethical OR AB ethical OR TI religiosity OR AB religiosity OR TI religion OR AB religion OR TI spirituality OR AB spirituality OR TI spiritual OR AB spiritual OR MA cross cultural comparison OR TI cross-cultural comparison OR AB cross-cultural comparison OR TI cross-cultural comparisons OR AB cross-cultural comparisons OR MA cultural characteristics OR TI cultural characteristics OR AB cultural characteristics OR MA cultural diversity OR TI cultural diversity OR AB cultural diversity OR MA cultural competency OR TI cultural competency OR AB cultural competency OR MA culture OR TI culture OR AB culture OR MA goals)

**Cinahl:**

(MH "patient care plans" OR MH "advance care planning" OR TI advance care planning OR AB advance care planning OR TI advance health care planning OR AB advance health care planning OR TI advance medical planning OR AB advance medical planning OR TI patient care planning OR AB patient care planning OR TI advanced medical planning OR AB advanced medical planning OR TI advanced care planning OR AB advanced care planning OR TI advanced health care planning OR AB advanced health care planning)

AND

(MH spirituality OR MH "Religion and Religions" OR TI ethics OR AB ethics OR TI ethical issues OR AB ethical issues OR TI ethical issue OR AB ethical issue OR TI moral policy OR AB moral policy OR TI moral policies OR AB moral policies OR TI ethical OR AB ethical OR TI religiosity OR AB religiosity OR TI religion OR AB religion OR TI spirituality OR AB spirituality OR TI spiritual OR AB spiritual OR MH "Ethnological Research" OR TI cross-cultural comparison OR AB cross-cultural comparison OR TI cross-cultural comparisons OR AB cross-cultural comparisons OR TI cultural characteristics OR AB cultural characteristics OR MH "cultural diversity" OR TI cultural diversity OR AB cultural diversity OR MH "cultural competence" OR TI cultural competency OR AB cultural competency OR MH culture OR TI culture OR AB culture OR MH "goals and objectives")

NOTE: MeSH “patient care planning” was replaced by CINAHL Heading “patient care plans”; “Religion” was replaced by “Religion and Religions”; “Cross-Cultural Comparison” was replaced by “Ethnological Research”; “Cultural Characteristics” does not exist in CINAHL Headings; “Cultural Competency” was replaced by “Cultural Competence”; “goals” does not exist in CINAHL Headings, so was replaced by “Goals and objectives”.
