# Supplementary material for: Underlying goals of advance care planning (ACP): a qualitative analysis of the literature
Source: BMC Palliat Care. 2020 Mar 6;19:27. doi: 10.1186/s12904-020-0535-1 (PMC7059342; doi:10.1186/s12904-020-0535-1)
Supplement: Supplementary file 2 — Additional file 2:. Codes per underlying goal. [file 12904_2020_535_MOESM2_ESM.docx]

**Additional file 2: codes per underlying goal**

**Respecting individual patient autonomy**

- *Codes for goals (n = 18; quotations: n = 245)*: [D decisions that reflect patient preferences] [D document preferences / completion of advance directives] [D improve applicability of advance directives] [D making advance decisions] [F appoint surrogate decision maker] [F describe role and duties of surrogate] [F determine who might help surrogate] [F protect surrogates] [H protect health care providers / decrease liability risk] [P choose treatments that the patient desires] [P control future actions of others] [P extend self-determination] [P giving/withholding informed consent for future treatment] [P increase patient sense of control] [P influence/guide future health care decisions that will be made when the patient will be incapacitated] [P reduce treatments that the patient did not want] [P respect individual self-determination] [P safeguard against doctors 'playing God']
- *Codes for objections (n = 42; quotations: n = 281)*: [O autonomy does not lead to a good death / good end of life care] [O based upon radical pluralism] [O cause harm / unintended consequences] [O causes confusion] [O damages patient-physician relationship] [O decisions are not made in isolation] [O denies interdependency and the importance of relational networks] [O denies that decision-making is engaged and emotional] [O difficult/impossible to consider all implications] [O disconnect between theory and practice] [O does not take into account individual patient needs] [O does not take into account justice] [O focused on American ethics] [O future interests are subordinated to former interests] [O health care professionals do not act upon wishes] [O inaccessibility of AD's] [O increased burden on health care professionals] [O increased burden on surrogates] [O invalid underlying assumptions] [O low completion rates of AD's] [O might assist in attempting suicide] [O narcissistic behavior] [O negative effects on family-health care professional relation] [O negative effects on patient-family relationships] [O negative effects on the community] [O not necessary / irrelevant] [O not necessary because surrogates will act] [O patients want surrogates to have leeway] [O patients will never have enough knowledge] [O people are not autonomous / detached from others] [O possible interference with physician's autonomy and sound medical care] [O predicting future preferences is difficult] [O preference to rely on others] [O preferences change over time] [O preferences change with progression of disease] [O preferences of questionable validity] [O promises more control than is possible] [O reasons behind preferences change, which makes former preferences not attributable] [O surrogate may make better decisions without ACP] [O there are not so many choices/options for patients] [O too general for clinical decision making] [O too much focus on legal status / checklist]

**Improving quality of care**

- *Codes for goals (n = 48; quotations: n = 338)*: [D avoid crisis decision making] [D decisions based on a set of considerations including context] [D decisions that are 'best' for all] [D decisions that are 'best' for the patient] [D decisions that are ethically sound] [D facilitate decision making] [D facilitate surrogate decision making] [D handling the risk of uncertainty / measured flexibility in responding to clinical situations] [D improve decision making] [F make family understand the patient's illness] [F prepare surrogate decision maker] [H decrease emotionality of discussing end-of-life issues] [H facilitate decision making for health care professionals] [H physician empowerment] [H reduce burden on health care providers] [H remind health care professionals to value patient involvement] [H service provision] [H standardise practice / organizational maintenance] [M achieve a good death] [M avoid prolongation of death] [M composed care culture / reduce stress among fellow residents] [M continuity of care] [M guide treatment] [M health-care centered outcomes] [M high-quality personalized care for incapacitated patients] [M improve quality of care] [M increase comfort] [M increase hospice use] [M less pain] [M planning for death] [M planning of care] [M prerequisite to patient-centered care] [M reduce undertreatment] [M set goals for medical care] [M timing of do-not-resuscitate orders] [M timing of palliative care] [P anticipate and consider aspects of which the patient was not aware] [P avoid medical outcomes that the patient deems unacceptable] [P concordance of treatments with patient preference] [P die in a way that is acceptable to the patient] [P discuss preferences for care] [P enhance choice] [P increase patient involvement in care] [P patient preferences known] [P patient preferences known to health care provider] [P patient preferences known to surrogate] [P patients deserve a say in treatment] [P prepare for future decision making]
- *Codes for objections (n = 13; quotations: n = 118)*: [O cultural taboo against speaking about death] [O discordance between surrogate decision and patient preference] [O does not appeal to everyone] [O increased burden on patient] [O one should be free to die without a prior plan (individual self-determination)] [O only for the convenience of health care professionals / health care system] [O people avoid thinking about end-of-life / people consider it too early] [O questionable cost-effectiveness / costs too much time or money] [O reduces hope] [O risk of pseudo-participation] [O risk to be regarded as correct and appropriate / moral imperative] [O too much focus on interventions] [O undermines autonomy]

**Strengthening relationships**

- *Codes for goals (n = 23; quotations: n = 130)*: [C decrease conflict] [C decrease conflict between health care provider and family members] [C decrease cross-cultural or intergenerational tensions] [C enhance commitment to the patient] [C enhance trust] [C honoring/strengthening relationships] [C improve communication between patient and health care provider] [C increase communication / breaking taboos] [C increase communication between health care provider and family] [C increase communication with loved ones] [C more agreement between family and health care provider] [C more agreement between patient and health care provider] [C resolve ethical conflicts] [C shared experiences] [C social functions] [F address needs of the family] [F family empowerment] [F help family] [F increase awareness among young people of acting as a surrogate] [F more agreement among family members] [F protect and legitimize nontraditional kinship relationships] [H reduce conflict within healthcare team] [P increase relational autonomy]
- *Codes for objections (n = 2; quotations: n = 4)*: [O fear that children take over too early] [O too much focus on relatives]

**Preparing for end-of-life**

- *Codes for goals (n = 38; quotations: n = 360)*: [F emotional preparation of loved ones for death and loss] [F increase family satisfaction with care] [F increase surrogate's confidence in decision-making] [F reduce cost for patient/family] [F reduce depressive symptoms] [F reduce family stress / relief of family burden] [F reduce feelings of guilt and regret] [M healthy way of life] [M improve quality of life] [P accept one's situation] [P address needs of the patient] [P aid/protect personal integrity] [P anticipated self-interpretation] [P being respected / psychological concerns addressed] [P clarify patient's questions, fears and values / defining quality of life] [P enhance 'the essence of being'] [P enhance patient experience / increase satisfaction] [P experience gerotranscendence] [P explore social factors that influence wellbeing] [P express personal values and goals] [P facing death / guided exploration of one's mortality] [P increase patient knowledge about the dying process] [P inform patients] [P maintain dignity] [P maintain/enhance hope] [P make patients understand their illness] [P overcoming aversion and facing decisions about dying] [P patient empowerment] [P prepare for death and dying / prepare for functional decline] [P preserve one's personal narrative] [P protect interests of older people] [P psychological/spiritual well-being / peace of mind / positive attitude] [P raise patient awareness about having options to choose from] [P reduce decisional conflict] [P reduce feelings of guilt and regret for patient] [P relief from depression] [P relief of anxiety and stress] [P time to adjust expectations / time to get used to situation]
- *Codes for objections (n = 2; quotations: n = 18)*: [O diverts attention from other important domains] [O risk to be regarded as a panacea]

**Reducing overtreatment**

- *Codes for goals (n = 7; quotations: n = 59)*: [M avoid inappropriate use of resources] [M limitation of resuscitation] [M limitations of medical treatments] [M reduce cost] [M reduce hospitalization] [M reduce ICU stay] [M reduce overtreatment]
- *Codes for objections (n = 6; quotations: n = 64)*: [O contrary to focusing on living / against nature] [O distrust of health care system] [O God determines / accept the mandate of nature] [O political goals / political correctness] [O pressure to refuse treatment] [O risk to be regarded as death panels]

**General**

- *Codes for goals (n = 7; quotations: n = 35)*: [G acceptable / no serious adverse effects] [G has legal force] [G multifaceted] [G no alternative available] [G part of our way of life] [G serves patients and families well] [G thought of as a good idea]
- *Codes for objections (n = 5; quotations: n = 57)*: [O barriers to successful implementation] [O complicated] [O conceptual ambiguity] [O evidence for benefit is inconclusive] [O preferred effect differs from patient to patient]
